# Fecal carriage and factors associated with extended-spectrum β-lactamase-producing Enterobacteriaceae among pregnant women at the tertiary referral hospital, Tanzania

**DOI:** 10.1186/s41182-020-00271-2

**Published:** 2020-10-08

**Authors:** Ambele M. Mwandigha, Doreen Kamori, Upendo O. Kibwana, Salim Masoud, Joel Manyahi, Mtebe Majigo

**Affiliations:** Department of Microbiology and Immunology, Muhimbili University of Health and Allied Sciences, P.O. Box 65001, Dar es Salaam, Tanzania

**Keywords:** Extended-spectrum beta-lactamase, Enterobacteriaceae, Fecal carriage, Antimicrobial resistance, Pregnancy

## Abstract

**Background:**

Infections due to extended-spectrum β-lactamase-producing Enterobacteriaceae (ESBL-E) are increasing worldwide. Evidence indicates that fecal carriage of ESBL-E in pregnancy predisposes women to potential life-threatening urinary tract infections and subsequently increasing the risk of neonatal infections. There is limited data regarding fecal carriage of ESBL-E and associated factors among pregnant women in Tanzania. We aimed to address the gap by determining the proportion of pregnant women with ESBL-E fecal carriage and identify the related factors.

**Methodology:**

A hospital-based cross-sectional study was conducted at Muhimbili National Hospital in Dar es Salaam, Tanzania. A total of 182 pregnant women at the gestational age of 37 weeks and above were enrolled. Participants’ socio-demographic, clinical, and hygienic information were collected by using a well-structured questionnaire. Rectal swabs were collected and processed for isolation of ESBL-E. The extended-spectrum β-lactamase production and antibiotic susceptibility test (AST) were performed using a double-disc synergy test and Kirby-Bauer disc diffusion method, respectively.

**Results:**

A total of 117 (64.3%) pregnant women were found to carry ESBL-E. Factors such as self-prescription of antibiotic medication during pregnancy, low education level, and toilet sharing were independently associated with ESBL-E fecal carriage. Five ESBL-E species that were isolated include *Escherichia coli* (84.6%), *Klebsiella pneumoniae* (8.9%), *Klebsiella oxytoca* (3.3%), *Citrobacter* spp. (1.6%), and *Enterobacter* spp. (1.6%). ESBL-E isolates demonstrated high resistance to aztreonam and sulphamethoxazole-trimethoprim.

**Conclusion:**

This study has revealed a relatively high fecal carriage of ESBL-E among pregnant women, suggesting that there is a need for routine screening among that population. We recommend further studies to explore comprehensively the factors associated with high fecal carriage of ESBL-E in pregnancy and the potential transmission kinetics to their newborn babies.

## Background

Antimicrobial resistance (AMR) is an emerging challenge in humans worldwide [1]. Extensive use of antibiotics in humans, animals, and plants may induce continuous and dynamic mutations to the bacteria [2]. Mutations can result in the production of resistant genes such as those encoding for extended-spectrum β-lactamase (ESBL) enzymes, which confer multidrug resistance [3]. Previous studies have reported the fecal carriage of ESBL-producing Enterobacteriaceae (ESBL-E) to range from 3.2 to 67.9% [4–8]. Around 110 million individuals in Africa are estimated to be ESBL-E carriers, which is attributed to high population density, disadvantaged access to drinking water, and poverty [9]. A study conducted in Tanzania reported ESBL-E fecal carriage of 11.6% among healthy children that resulted from extensive use of antibiotics [10].

Globally, the fecal carriage rate of ESBL-E among pregnant women is reported to range from 7.3 to 15.4%, which is comparable to the healthy population in different regions [11–13]. In Africa, ESBL-E fecal carriage average is 17% among pregnant women [14] and 15% in post-delivery women [15]. The main concern of ESBL-E fecal carriage in pregnancy is the increased risk of transmission of these resistant strains to newborn babies that subsequently may contribute to neonatal sepsis [13, 16]. Also, ESBL-E carriage increases the risk of transmission to health care providers and other hospitalized patients.

Furthermore, pregnant women are among the immunocompromised group, who are prone to different bacterial infections. The colonization of ESBL-E in the urinary tract of pregnant women due to fecal-vaginal transmission often results in urinary tract infection (UTI) [17, 18]. Globally, the proportion of pregnant women colonized by ESBL-E in the urinary tract ranges from 2.0 to 75%, the highest percentage found in developing countries [19, 20]. In Africa, a high proportion above 70% is seen in sub-Saharan countries due to extensive antibiotic use in agriculture, veterinary, and human health centers [14, 21]. In Tanzania, the proportion of ESBL-E to urinary isolates in pregnant women ranges between 18.8 and 45.2% [22].

In our setting, there is limited data on ESBL-E carriage among pregnant women. Therefore, this study determined the fecal carriage of ESBL-E among pregnant women, associated factors, and antimicrobial resistance patterns of ESBL-E bacteria isolates.

## Materials and methods

### Study design and settings

The study was a hospital-based cross-sectional design conducted from February to May 2019 at Muhimbili National Hospital (MNH) in Dar es Salaam, Tanzania. The MNH is the largest tertiary health care facility in Tanzania with 25 departments, 106 service units, and a 1500-bed capacity. The hospital serves as a research center, university teaching hospital, and a referral hospital. Around 1000 to 1200 outpatients attend different clinics per day with about 1000 to 1200 admissions per week. A total of 2700 employees, including 300 doctors, 900 nurses, and 1500 supporting staffs, are available at the facility. In this hospital, pregnant women attend at least four visits at an interval of 4 weeks, starting at 12-week gestational age. The clinic provides antenatal services to an average of 700 pregnant women per month, with approximately 25 pregnant women per clinic visit. On average, nearly a quarter of pregnant women attending per month have a gestational age equal to or greater than 37 weeks. The total number of deliveries at MNH from January to December 2019 was approximately 2126.

### Study population, sample size, and sampling procedure

The study enrolled pregnant women who were at the gestational age of 37 weeks and above attending the antenatal clinic and consented to participate. Pregnant women with known increased risks for bacterial colonization, such as gestational diabetes, history of prolonged hospital stay during pregnancy, malignancy, and renal and liver diseases, were not eligible for inclusion in the study.

The study used the Kish Leslie formula, *n* = *z*^2^ *p*(1 − *p*)/*e*^2^, to calculate the sample size. In the formula, “*n*” stands for the required minimum sample size and “*z*” is the value for the level of confidence (1.96) at a 95% confidence interval. The expected proportion (*p*) of ESBL-E among pregnant women was estimated at 18.5% from a study conducted in Madagascar [23], and the margin of error “*e*” was 6%. The minimum required sample size was estimated to be 161 pregnant women. Convenient sampling was used to recruit pregnant women in the study. New eligible pregnant women were consecutively enrolled during their clinic visit until the representative sample size was attained.

### Data collection

A well-structured questionnaire was used to collect socio-demographic information (age and level of education), history of self-prescription of antibiotic, and hygienic practices (the type of toilet, toilet sharing, handwashing, and use of treated drinking water). The antenatal card was the source of clinical information that included gestational age, number of parity, HIV status, and comorbidities.

### Study variables

Education level was classified as a low level for primary education and below and as a high level for secondary education and above. The self-prescription of antibiotic medication in pregnancy was defined as the use of any antibiotic without a clinician’s prescription during pregnancy at least once in the last 4 weeks. The type of toilet used was the one available at home in the previous 3 months. Toilet sharing in pregnancy meant the use of a common/public toilet at home used by more than one individual in the last 3 months. Handwashing with soap before eating was counted when hands were washed with soap every day during the main meals in the previous week. Handwashing with soap after defecation was defined when soap was used to wash hands after defecation every day in the last week. The use of treated drinking water was considered when treated water was used for drinking at least once a day in the previous week.

### Sample collection and laboratory procedures

Rectal swabs were collected from pregnant women by trained medical personnel. Briefly, the medical personnel inserted a sterile cotton wool swab in the rectum for about 10 s. Then, the collected specimen was kept into the Cary-Blair medium and then transported to the Central Pathology Laboratory (CPL) within 2 h after collection [24].

The rectal swab specimen was inoculated onto MacConkey agar (MCA), supplemented with 2.0 μg/ml ceftazidime and incubated aerobically at 37 °C overnight [25]. Preliminary identification of isolates was based on colonial morphology and Gram staining properties. All isolates were sub-cultured on nutrient agar (NA) and incubated aerobically at 37 °C overnight. Isolates from NA were identified using conventional biochemical tests. The identification tests included the oxidase test, Kligler Iron Agar, Sulfur-Indole-Motility test, Simmons’ citrate test, and urease test, as previously described [15]. Isolates were confirmed as ESBL-E by a double-disc synergy test [26].

Five antibiotic discs: gentamicin (10 μg), aztreonam (30 μg), sulphamethoxazole-trimethoprim (1.25/23.75), meropenem (10 μg), and ciprofloxacin (5 μg), were used for the antibiotic susceptibility test by using the Kirby-Bauer disc diffusion method. Except for aztreonam, the remained discs were chosen because they were reported to be susceptible to ESBL-E [15, 27, 28], whereas aztreonam was tested for susceptibility to check if all ESBL-E isolates are either resistant to aztreonam or not. The resistance pattern was defined based on the Clinical and Laboratory Standard Institute (CLSI) guidelines of 2018.

### Quality control

All reference organisms and reagents were clearly and uniquely labeled, dated, and stored at optimal conditions. The operating temperatures of the refrigerator and incubator were monitored and documented daily. All culture media were prepared following the manufacturer’s guidelines and internal standard operating procedures and were tested for performance and sterility. We standardized the turbidity of isolate suspension with a 0.5 McFarland standard. As per CLSI (2018), *Klebsiella pneumoniae* (ATCC-700603) and *Escherichia coli* (ATCC-25922) were the positive and negative control bacteria strains, respectively [29].

### Statistical analysis

STATA version 15.1 software was used for statistical analysis. Continuous variables were summarized as the median and interquartile range (IQR), whereas proportions were used to describe categorical variables. Group differences were examined by using Fisher’s exact test for categorical variables and *t* test for continuous variables. Binary logistic regression was performed to identify factors associated with ESBL-E fecal carriage among pregnant women depending on the crude odds ratio (COR) with a 95% confidence interval (CI). Multivariable logistic regression was performed to examine the associations between the outcome variable and independent variables after adjustment of all the factors that showed significance in the bivariate analysis, such as education level, self-prescription of antibiotic medication during pregnancy, toilet sharing, and handwashing with soap after defecation. Associations in the multivariable logistic models were presented as adjusted odds ratios (AOR) with 95% CI. A two-sided *p* value < 0.05 was considered significant.

## Results

### Characteristics and hygienic practices of the study participants

A total of 182 pregnant women with a median age of 29 years, IQR [25–32], with gestational age, and parity of 39 weeks, IQR [40–38], and 2, IQR [3–1], respectively, were enrolled in the study. Out of 182, 56.0% had a high level of education, and 95.0% were HIV seronegative. Furthermore, 52.2% (95/182) of the participants reported self-prescription of antibiotic medication during pregnancy (Table 1). A total of 154 (84.6%) participants were using flush toilets, whereby 86 (74.7%) reported sharing toilets. Concerning hygiene practices, the majority (87.4%) reported washing hands using soap before eating. Likewise, 85.7% affirmed washing hands using soap after defecation, and 62.1% reported the use of treated drinking water (Table 1).

**Table 1:** Distribution of socio-demographic, clinical, and hygienic variables among pregnant women (*N* = 182)

Variable	Total number	Percentage (%)
**Age**		
18–25	50	27.5
26–35	107	58.8
36+	25	13.7
**Gestational age**		
37–39	121	66.5
40–42	57	31.3
43–45	4	2.2
**Number of parity**		
0–1	73	40.1
2	53	29.1
3+	56	30.8
**Educational level**		
Low	80	44.0
High	102	56.0
**HIV status**		
Positive	9	5.0
Negative	173	95.0
**Self-prescription of antibiotic medication in pregnancy**		
Yes	95	52.2
No	87	47.8
**Type of toilet used**		
Flush	154	84.6
Hole	28	15.4
**Toilet sharing**		
No	46	25.3
Yes	136	74.7
**Handwashing with soap before eating**		
Yes	159	87.4
No	23	12.6
**Handwashing with soap after defecation**		
Yes	156	85.7
No	26	14.3
**Use of treated drinking water**		
Yes	113	62.1
No	69	37.9

### Fecal carriage of ESBL-E

The fecal carriage of ESBL-E was detected in 117 (64.3%) pregnant women. Participants with a history of self-prescription of antibiotic medication during the current pregnancy were more colonized with ESBL-E than those without a history of self-prescription of antibiotic medication (*p* < 0.001). Not washing hands with soap after defecation and toilet sharing were associated with higher fecal carriage of ESBL-E (*p* < 0.05). We observed a trend of decrease in the proportion of ESBL-E fecal carriage with an increase in participants’ education level. The difference between low (77.5%) and high (53.9%) education levels was significant (*p* = 0.001). The differences of ESBL-E fecal carriage between the categories among age groups, gestational age, parity, HIV status, types of toilet used, handwashing with soap before eating, and use of treated water was not significant (*p* > 0.05) (Table 2).

**Table 2:** Fecal carriage of ESBL-E among pregnant women by social-demographic, clinical factors, and hygienic factors

Variable	Total number	Fecal carriage, ***n*** (%)	***p*** value
Overall	182	117 (64.3)	
**Age group**			0.06
18–25	50	39 (78.0)	
26–35	107	63 (58.9)	
35+	25	15 (60.0)	
**Gestational age**			0.69
37–39	121	75 (62.0)	
40–42	57	39 (68.4)	
43–45	4	3 (75.0)	
**Number of parity**			0.26
0–1	73	50 (68.5)	
2	53	36 (67.9)	
3+	56	31 (55.4)	
**Education level**			**0.001**
Low	80	62 (77.5)	
High	102	55 (53.9)	
**HIV status**			0.16
Positive	9	8 (88.9)	
Negative	173	109 (63.0)	
**Self-prescription of antibiotic medication in pregnancy**			**0.001**
Yes	95	72 (75.8)	
No	87	45 (51.7)	
**Types of toilet used**			1.00
Flush	154	99 (64.3)	
Hole	28	18 (64.3)	
**Toilet sharing**			**0.01**
No	46	22 (47.8)	
Yes	136	95 (69.9)	
**Handwashing with soap before eating**			0.17
Yes	159	99 (62.3)	
No	23	18 (78.3)	
**Handwashing using soap after defecation**			**0.03**
Yes	156	95 (60.9)	
No	26	22 (84.6)	
**Use of treated drinking water**			0.27
Yes	113	69 (61.1)	
No	69	48 (69.6)	

In bold *p* value of less than 0.05 that indicates statistically significant association (Fisher’s exact test)

### Predictors of ESBL-E fecal carriage

By using bivariate analysis, participants with low education level were observed to have three times more odds of ESBL-E fecal carriage than participants with high education level (cOR 2.94, 95% CI 1.53–5.66). The self-prescription of antibiotic medication during pregnancy had an increased probability of ESBL-E fecal carriage (cOR, 2.92, 95% CI 1.56–5.49). Toilet sharing and hand washing after defecation had 2.5 and 3.5 times the odds of ESBL-E fecal carriage, respectively (Table 3).

**Table 3:** Socio-demographic, clinical, and hygienic factors associated with ESBL-E fecal carriage by bivariate analysis

Variable	Total number	Positive, ***n*** (%)	cOR	95% CI	***p*** value
**Age (*p*** **= 0.06)**	182	117 (64.3)	0.95	0.89–1.00	0.07
**Educational level (*p*** **= 0.001)**					
Low	80	62 (77.5)	2.94	1.53–5.66	**0.001**
High	102	55 (53.9)	1		
**HIV status (*p*** **= 0.16)**					
Positive	9	8 (88.9)	4.70	0.57–38.42	0.15
Negative	173	109 (63.0)	Ref		
**Self-prescription of antibiotic medication in pregnancy (*p*** **= 0.001)**					
Yes	95	72 (75.8)	2.92	1.56–5.49	**0.001**
No	87	45 (51.7)	Ref		
**Toilet sharing (*p*** **= 0.01)**					
No	46	22 (47.8)	Ref		
Yes	136	95 (69.9)	2.53	1.27–5.01	**0.008**
**Handwashing with soap before eating (*p*** **= 0.17)**					
Yes	159	99 (62.3)	Ref		
No	23	18 (78.3)	2.18	0.77–6.18	0.14
**Handwashing using soap after defecation (*p*** **= 0.03)**					
Yes	156	95 (60.9)	Ref		
No	26	22 (84.6)	3.53	1.16–10.74	**0.03**

*cOR* crude odds ratio, *Ref* reference

On multivariate analysis, the factors that were independently associated with ESBL-E fecal carriage were education level, self-prescription of antibiotic medication during pregnancy, and toilet sharing. The odds of carrying ESBL-E were two times higher among participants having a low education level than high education level (aOR = 2.19, 95% CI = 1.08–4.43). Participants with a history of self-prescription of antibiotic medication had three times higher probability of carrying ESBL-E (aOR = 3.11, 95% CI = 1.59–6.01). The odds of carrying ESBL-E was two times higher among those who shared toilets during their pregnancy (aOR = 2.39, 95% CI = 1.10–5.21) (Table 4).

**Table 4:** Multivariate logistic regression for the factors independently associated with ESBL-E fecal carriage

Variable	ESBL-E fecal carriage, ***n*** (%)	cOR	95% CI	***p*** value	aOR	95% CI	***p*** value
**Education level**							
Low	62 (77.5)	2.94	1.53–5.66	**0.001**	2.19	1.08–4.43	**0.04**
High	55 (53.9)	Ref			Ref		
**Self-prescription of antibiotic medication in pregnancy (*p*** **= 0.001)**							
Yes	72 (75.8)	2.92	1.56–5.49	**0.001**	3.11	1.59–6.01	**0.001**
No	45 (51.7)	Ref			Ref		
**Toilet sharing (*p*** **= 0.01)**							
No	22 (47.8)	Ref			Ref		
Yes	95 (69.9)	2.53	1.27–5.01	**0.008**	2.39	1.10–5.21	**0.03**
**Handwashing using soap after defecation (*p*** **= 0.03)**							
Yes	95 (60.9)	Ref			Ref		
No	22 (84.6)	3.53	1.16–10.74	**0.03**	2.69	0.83–8.72	0.10

*cOR* crude odds ratio, *aOR* adjusted odds ratio, *Ref* reference

### ESBL-E species and antimicrobial resistance patterns

**Fig. 1:**
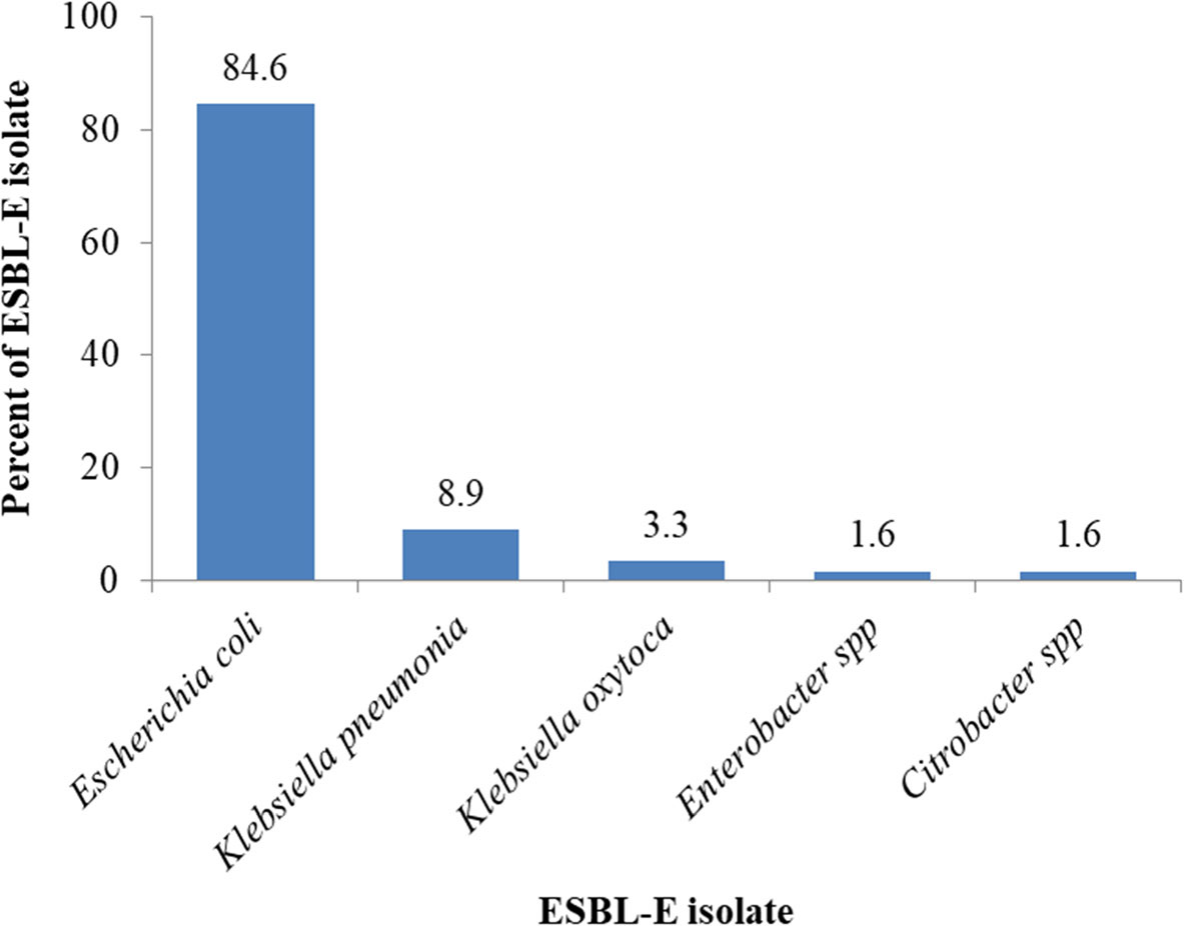
Proportion of ESBL-E species isolated from pregnant women. The figure illustrates the distribution of specific ESBL-E isolates obtained from 182 pregnant women at Muhimbili National Hospital

Five bacterial species were identified among 123 isolates. *Escherichia coli* was the most predominant with a proportion of 84.6%, followed by *Klebsiella pneumoniae* (8.9%), *Klebsiella oxytoca* (3.3%), *Citrobacter* spp. (1.6%), and *Enterobacter* spp. (1.6%) (Fig. 1).

The antibiotic susceptibility test was conducted using five drugs. Aztreonam was highly resisted by *Citrobacter* spp. (100.0%), *K. pneumoniae* (90.9%), *E. coli* (85.6%), and *K. oxytoca* (75.0%). High resistance to sulphamethoxazole-trimethoprim was shown by *Enterobacter* spp. (100.0%), *E. coli* (76.0%), *K. oxytoca* (75.0%), and *K. pneumoniae* (63.6%). Resistance to ciprofloxacin was the lowest ranging from 0 to 25%. Among ESBL-E isolates, *K. oxytoca* was found to have the highest resistance to most antibiotics tested, while *Citrobacter* spp. had the least resistance compared to all other isolates (Table 5).

**Table 5:** Antimicrobial resistance patterns among ESBL-E isolates

ESBL-E isolates	Gentamicin, ***n*** (%)	Aztreonam, ***n*** (%)	Sulphamethoxazole-trimethoprim, ***n*** (%)	Meropenem, ***n*** (%)	Ciprofloxacin, ***n*** (%)
***E. coli*** **(*N*** **= 104)**	1 (25.0)	89 (85.6)	79 (76.0)	31 (29.8)	24 (23.1)
***K. pneumoniae*** **(*N*** **= 11)**	4 (36.4)	10 (90.9)	7 (63.6)	3 (27.3)	1 (9.1)
***K. oxytoca*** **(*N*** **= 4)**	18 (17.3)	3 (75.0)	3 (75.0)	3 (75.0)	1 (25.0)
***Enterobacter*** **spp. (*N*** **= 2)**	0	1 (50)	2 (100.0)	0	0
***Citrobacter*** **spp. (*N*** **= 2)**	0	2 (100.0)	1 (50)	0	0

## Discussion

This study revealed a high fecal carriage of ESBL-E among pregnant women, which coincide with findings by Djuikoue et al. in Cameroon that reported the fecal carriage of 66.3% in a similar population [30]. In contrast, other studies have reported a lower fecal carriage compared to our findings. For example, Fortin et al. in Nigeria reported the fecal carriage of 31.7% [31], Chereau et al. in Madagascar reported 18.6% [23], and Nelson et al. in Mwanza, Tanzania reported 15% [15]. Several factors may attribute to the high ESBL-E fecal carriage rate reported in this study, including misuse of antibiotics, low level of education, and poor hygienic practices.

The majority of women with ESBL-E fecal carriage had a low level of education, indicating an increased risk of ESBL-E fecal carriage with a low level of education; which is consistent with results from other previous studies [17, 30]. The low level of education could be accompanied by a lack of knowledge that predisposes these women to misuse of antibiotics and poor hygienic practices. These factors have been previously reported to be associated with ESBL-E carriage [11, 26–28].

We observed that the irrational use of antibiotics during pregnancy was significantly associated with ESBL-E carriage. The participants who had self-prescription of antibiotic medication had an increased chance of ESBL-E fecal carriage. This finding is comparable with other studies that have demonstrated an association between prior antibiotic exposure and carriage or infection with ESBL-E [15, 29, 31]. In this study, pregnant women who reported sharing toilets with others had a high risk of carrying ESBL-E. The increased risk might be influenced by poor toilet cleanliness and self-hygienic practices, which create a high chance of transmission of resistant pathogens [32–34].

In the present study, we observed that ESBL-E had low and moderate resistance to ciprofloxacin and gentamicin as well as meropenem, respectively. However, ESBL-E demonstrated high resistance to aztreonam and sulphamethoxazole-trimethoprim. These findings concur with other studies [15, 35, 36]. With the current status of an increase in antimicrobial resistance, our findings emphasize the need to perform susceptibility tests for ESBL pathogens to provide guidance on the drug of choice for treatment in our setting.

Our study may have some limitations. First, the findings from hospital-based design cannot be generalized to the community as there might be selection bias. The study recruited pregnant women at the last visit, so there is a possibility that these participants contacted ESBL-E during other antenatal visits rather than from the community. Second, we acknowledge that reliance on self-reported information may have introduced the recall bias; because some questions are required to recall back as far as 3 months. Thirdly, the study was not able to determine the seasonality of ESBL-E prevalence. Lastly, the study was conducted in a tertiary hospital where there is an increase in accessibility of antibiotics in the urban area compared to suburban and rural areas; hence, the present findings cannot be generalized.

## Conclusion

The fecal carriage of ESBL-E among pregnant women is relatively high, and most of the isolates are resistant to some antibiotics commonly used in our settings. We observed that prior exposure to antibiotics increases the possibility of ESBL-E carriage. The findings indicate that antimicrobial stewardship is essential for checking the susceptible drugs for treating ESBL-E-associated infections. Our findings suggest a need to undertake comprehensive precaution measures by providing health education to the health care providers and patients on the prevention of nosocomial transmission. In light of these findings, more studies are needed to determine further the risk of transmission to the newborns from mothers who are colonized by ESBL-E and determine the antibiotic profile for ESBL-producing bacteria.

## Data Availability

All relevant data generated and analyzed during this study are available from the corresponding author on reasonable request.

## Abbreviations

AMR: Antimicrobial resistance
ANC: Antenatal clinic
AST: Antibiotic susceptibility test
CLSI: Clinical Laboratory Standard Institute
DDST: Double-disc synergy test
ESBL: Extended-spectrum beta-lactamase
ESBL-E: Extended-spectrum beta-lactamase-producing Enterobacteriaceae
HIV: Human immunodeficiency virus
MCA: MacConkey agar
MNH: Muhimbili National Hospital
MUHAS: Muhimbili University of Health and Allied Sciences
NA: Nutrient agar
UTI: Urinary tract infection
WHO: World Health Organization
